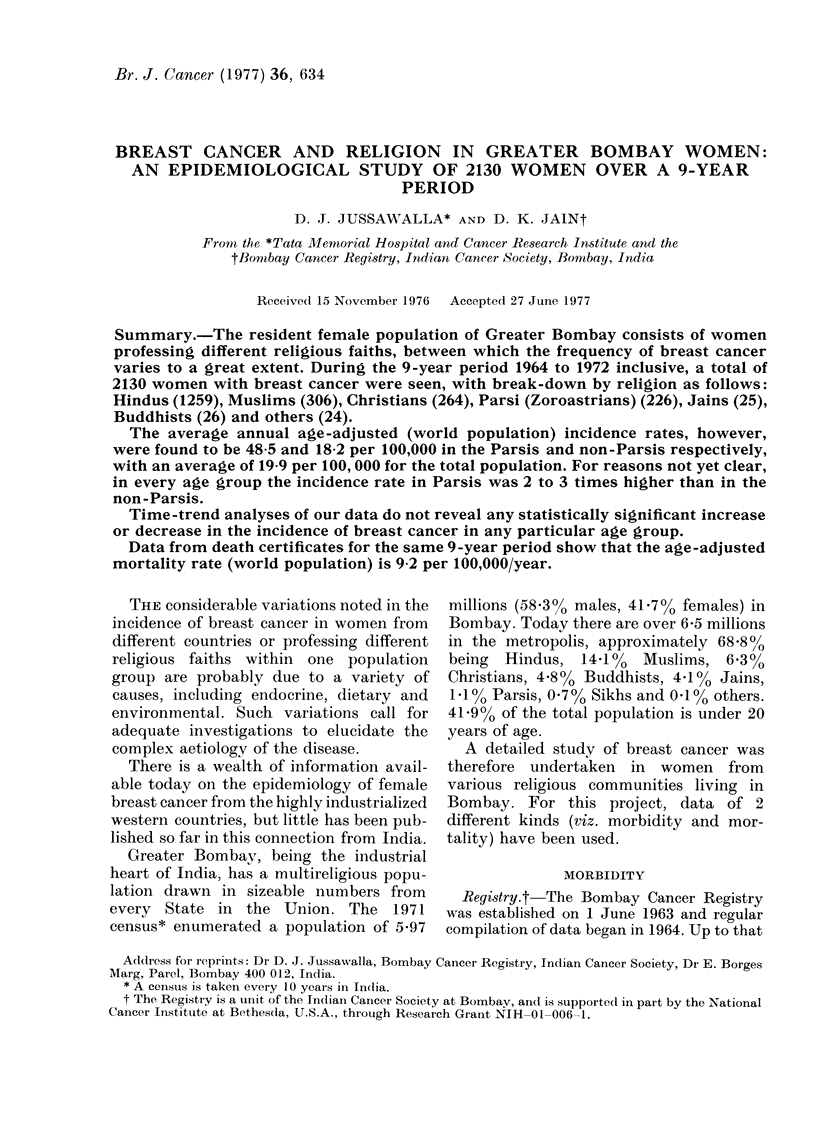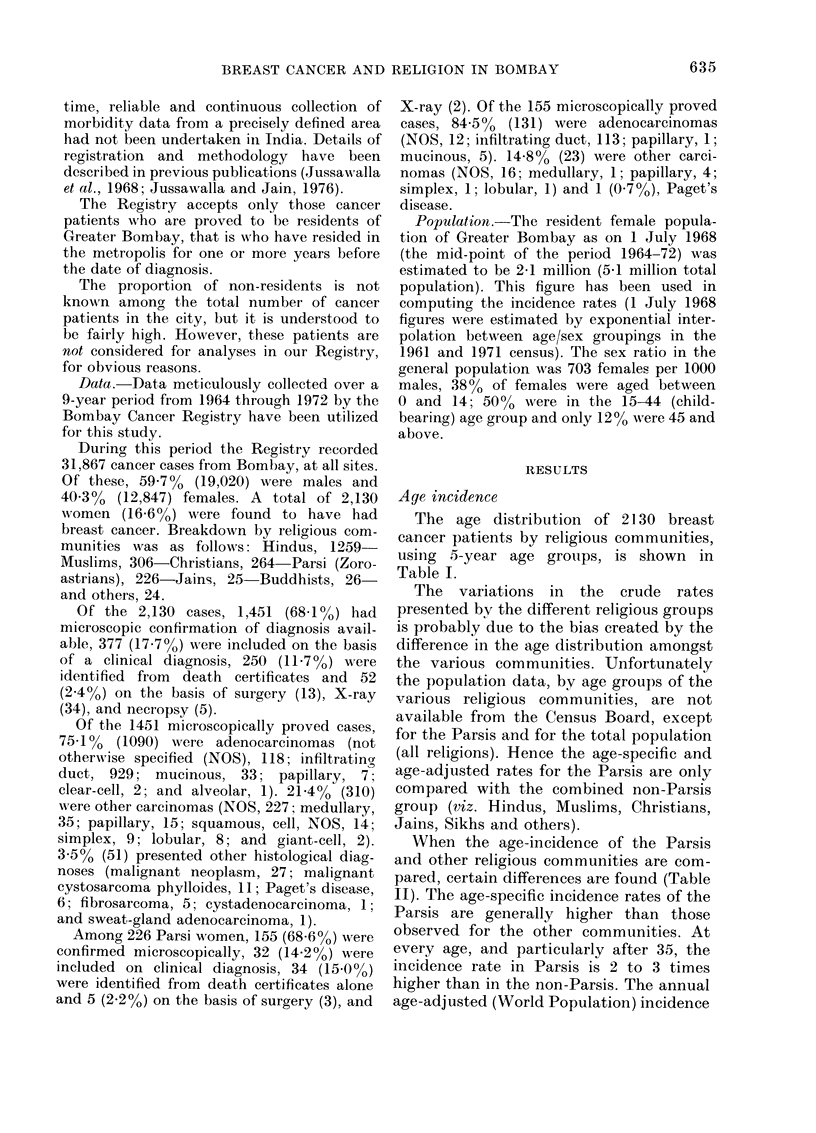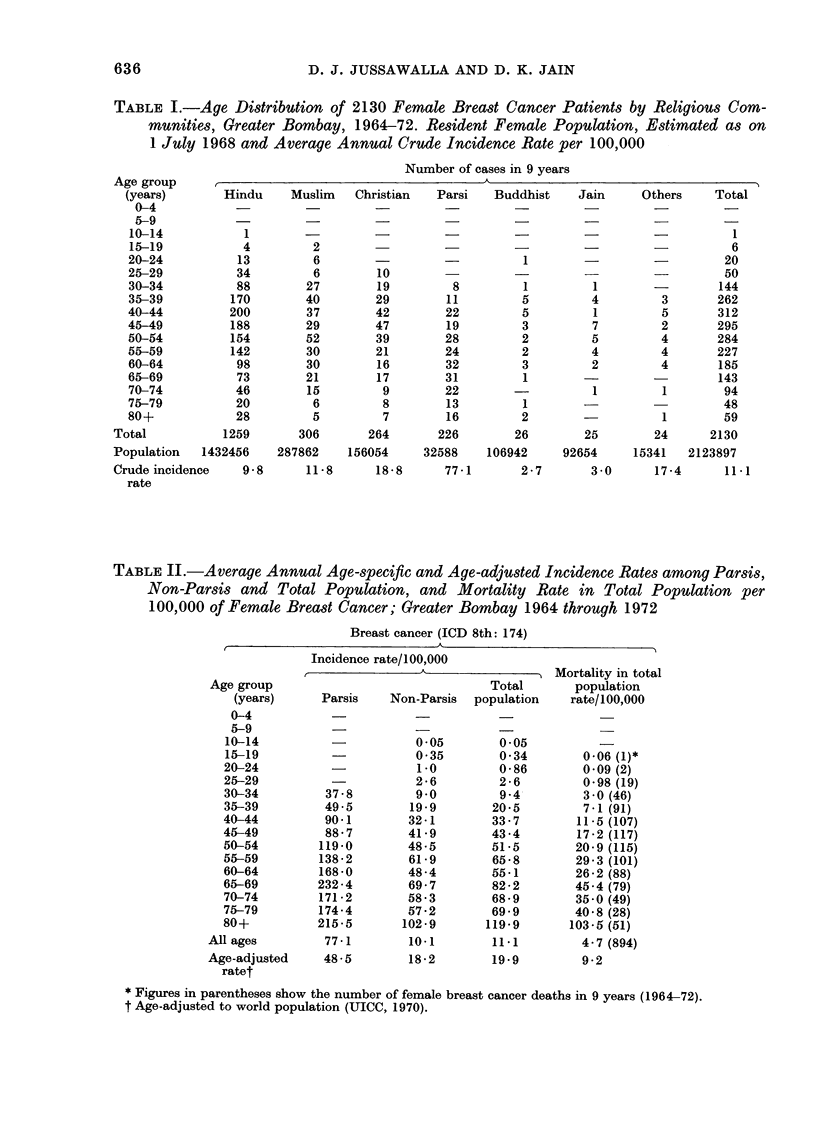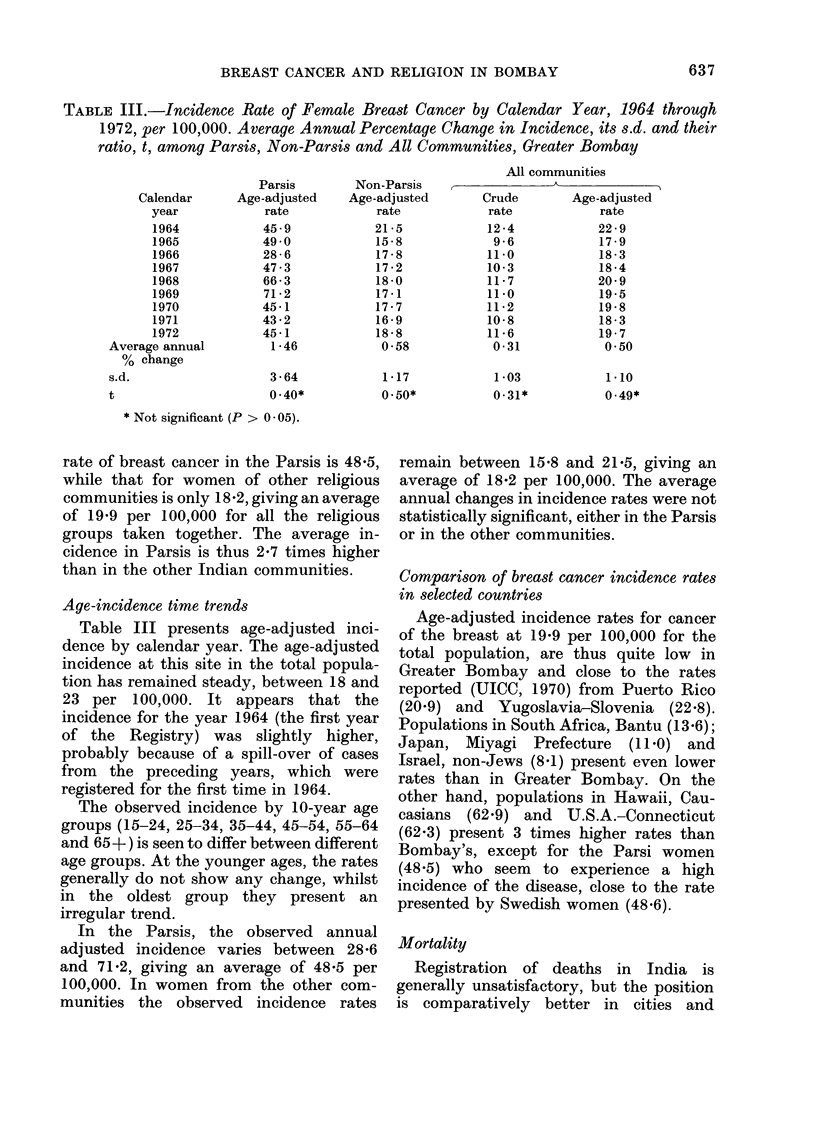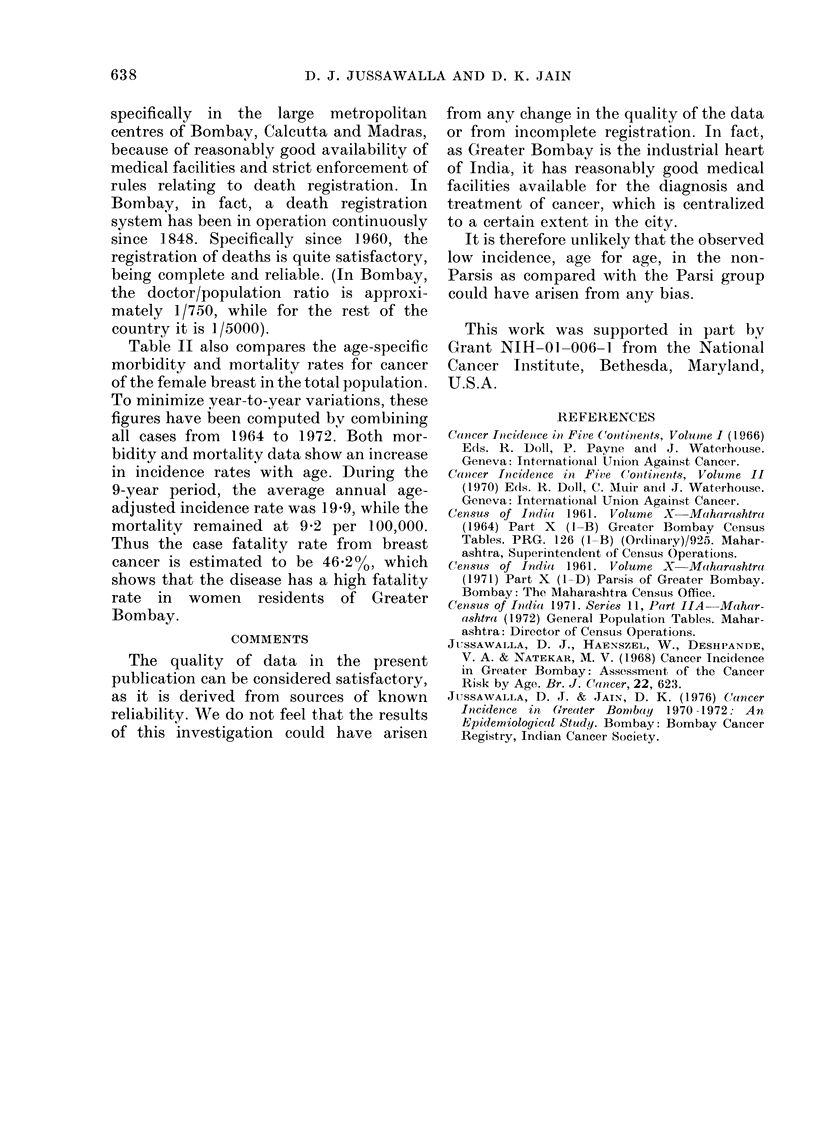# Breast cancer and religion in greater Bombay women: an epidemiological study of 2130 women over a 9-year period.

**DOI:** 10.1038/bjc.1977.241

**Published:** 1977-11

**Authors:** D. J. Jussawalla, D. K. Jain

## Abstract

The resident female population of Greater Bombay consists of women professing different religious faiths, between which the frequency of breast cancer varies to a great extent. During the 9-year period 1964 to 1972 inclusive, a total of 2130 women with breast cancer were seen, with break-down by religion as follows: Hindus (1259), Muslims (306), Christians (264), Parsi (Zoroastrians) (226), Jains (25), Buddhists (26) and others (24). The average annual age-adjusted (world population) incidence rates, however, were found to be 48.5 and 18.2 per 100,000 in the Parsis and non-Parsis respectively, with an average of 19.9 per 100,000 for the total population. For reasons not yet clear, in every age group the incidence rate in Parsis was 2 to 3 times higher than in the non-Parsis. Time-trend analyses of our data do not reveal any statistically significant increase or decrease in the incidence of breast cancer in any particular age group. Data from death certificates for the same 9-year period show that the age-adjusted mortality rate (world population) is 9.2 per 100,000/year.


*Br. J. Cancer* (1977) **36**, 634

### BREAST CANCER AND RELIGION IN GREATER BOMBAY WOMEN: AN EPIDEMIOLOGICAL STUDY OF 2130 WOMEN OVER A 9-YEAR PERIOD

D. J. JUSSAWALLA* AND D. K. JAIN†

*From the *Tata Memorial Hospital and Cancer Research Institute and the †Bombay Cancer Registry, Indian Cancer Society, Bombay, India*

Received 15 November 1976 Accepted 27 June 1977

**Summary.—The resident female population of Greater Bombay consists of women professing different religious faiths, between which the frequency of breast cancer varies to a great extent. During the 9-year period 1964 to 1972 inclusive, a total of 2130 women with breast cancer were seen, with break-down by religion as follows: Hindus (1259), Muslims (306), Christians (264), Parsi (Zoroastrians) (226), Jains (25), Buddhists (26) and others (24).**

**The average annual age-adjusted (world population) incidence rates, however, were found to be 48·5 and 18·2 per 100,000 in the Parsis and non-Parsis respectively, with an average of 19·9 per 100, 000 for the total population. For reasons not yet clear, in every age group the incidence rate in Parsis was 2 to 3 times higher than in the non-Parsis.**

**Time-trend analyses of our data do not reveal any statistically significant increase or decrease in the incidence of breast cancer in any particular age group.**

**Data from death certificates for the same 9-year period show that the age-adjusted mortality rate (world population) is 9·2 per 100,000/year.**

THE considerable variations noted in the incidence of breast cancer in women from different countries or professing different religious faiths within one population group are probably due to a variety of causes, including endocrine, dietary and environmental. Such variations call for adequate investigations to elucidate the complex aetiology of the disease.

There is a wealth of information available today on the epidemiology of female breast cancer from the highly industrialized western countries, but little has been published so far in this connection from India.

Greater Bombay, being the industrial heart of India, has a multireligious population drawn in sizeable numbers from every State in the Union. The 1971 census* enumerated a population of 5·97 millions (58·3% males, 41·7% females) in Bombay. Today there are over 6·5 millions in the metropolis, approximately 68·8% being Hindus, 14·1% Muslims, 6·3% Christians, 4·8% Buddhists, 4·1% Jains, 1·1% Parsis, 0·7% Sikhs and 0·1% others. 41·9% of the total population is under 20 years of age.

A detailed study of breast cancer was therefore undertaken in women from various religious communities living in Bombay. For this project, data of 2 different kinds (*viz.* morbidity and mortality) have been used.

### MORBIDITY

*Registry.*†—The Bombay Cancer Registry was established on 1 June 1963 and regular compilation of data began in 1964. Up to that

Address for reprints: Dr D. J. Jussawalla, Bombay Cancer Registry, Indian Cancer Society, Dr E. Borges Marg, Parel, Bombay 400 012, India.

* A census is taken every 10 years in India.

† The Registry is a unit of the Indian Cancer Society at Bombay, and is supported in part by the National Cancer Institute at Bethesda, U.S.A., through Research Grant NIH-01-006-1.

time, reliable and continuous collection of morbidity data from a precisely defined area had not been undertaken in India. Details of registration and methodology have been described in previous publications (Jussawalla *et al.*, 1968; Jussawalla and Jain, 1976).

The Registry accepts only those cancer patients who are proved to be residents of Greater Bombay, that is who have resided in the metropolis for one or more years before the date of diagnosis.

The proportion of non-residents is not known among the total number of cancer patients in the city, but it is understood to be fairly high. However, these patients are *not* considered for analyses in our Registry, for obvious reasons.

*Data.*—Data meticulously collected over a 9-year period from 1964 through 1972 by the Bombay Cancer Registry have been utilized for this study.

During this period the Registry recorded 31,867 cancer cases from Bombay, at all sites. Of these, 59·7% (19,020) were males and 40·3% (12,847) females. A total of 2,130 women (16·6%) were found to have had breast cancer. Breakdown by religious communities was as follows: Hindus, 1259—Muslims, 306—Christians, 264—Parsi (Zoroastrians), 226—Jains, 25—Buddhists, 26—and others, 24.

Of the 2,130 cases, 1,451 (68·1%) had microscopic confirmation of diagnosis available, 377 (17·7%) were included on the basis of a clinical diagnosis, 250 (11·7%) were identified from death certificates and 52 (2·4%) on the basis of surgery (13), X-ray (34), and necropsy (5).

Of the 1451 microscopically proved cases, 75·1% (1090) were adenocarcinomas (not otherwise specified (NOS), 118; infiltrating duct, 929; mucinous, 33; papillary, 7; clear-cell, 2; and alveolar, 1). 21·4% (310) were other carcinomas (NOS, 227; medullary, 35; papillary, 15; squamous, cell, NOS, 14; simplex, 9; lobular, 8; and giant-cell, 2). 3·5% (51) presented other histological diagnoses (malignant neoplasm, 27; malignant cystosarcoma phylloides, 11; Paget's disease, 6; fibrosarcoma, 5; cystadenocarcinoma, 1; and sweat-gland adenocarcinoma, 1).

Among 226 Parsi women, 155 (68·6%) were confirmed microscopically, 32 (14·2%) were included on clinical diagnosis, 34 (15·0%) were identified from death certificates alone and 5 (2·2%) on the basis of surgery (3), and X-ray (2). Of the 155 microscopically proved cases, 84·5% (131) were adenocarcinomas (NOS, 12; infiltrating duct, 113; papillary, 1; mucinous, 5). 14·8% (23) were other carcinomas (NOS, 16; medullary, 1; papillary, 4; simplex, 1; lobular, 1) and 1 (0·7%), Paget's disease.

*Population.*—The resident female population of Greater Bombay as on 1 July 1968 (the mid-point of the period 1964–72) was estimated to be 2·1 million (5·1 million total population). This figure has been used in computing the incidence rates (1 July 1968 figures were estimated by exponential interpolation between age/sex groupings in the 1961 and 1971 census). The sex ratio in the general population was 703 females per 1000 males, 38% of females were aged between 0 and 14; 50% were in the 15–44 (child-bearing) age group and only 12% were 45 and above.

### RESULTS

### *Age incidence*

The age distribution of 2130 breast cancer patients by religious communities, using 5-year age groups, is shown in Table I.

The variations in the crude rates presented by the different religious groups is probably due to the bias created by the difference in the age distribution amongst the various communities. Unfortunately the population data, by age groups of the various religious communities, are not available from the Census Board, except for the Parsis and for the total population (all religions). Hence the age-specific and age-adjusted rates for the Parsis are only compared with the combined non-Parsis group (*viz.* Hindus, Muslims, Christians, Jains, Sikhs and others).

When the age-incidence of the Parsis and other religious communities are compared, certain differences are found (Table II). The age-specific incidence rates of the Parsis are generally higher than those observed for the other communities. At every age, and particularly after 35, the incidence rate in Parsis is 2 to 3 times higher than in the non-Parsis. The annual age-adjusted (World Population) incidence

TABLE I.—*Age Distribution of* 2130 *Female Breast Cancer Patients by Religious Communities, Greater Bombay,* 1964–72. *Resident Female Population, Estimated as on* 1 *July* 1968 *and Average Annual Crude Incidence Rate per* 100,000

| Age group (years) | Number of cases in 9 years | | | | | | | |
|---|---|---|---|---|---|---|---|---|
| | Hindu | Muslim | Christian | Parsi | Buddhist | Jain | Others | Total |
| 0–4 | — | — | — | — | — | — | — | — |
| 5–9 | — | — | — | — | — | — | — | — |
| 10–14 | 1 | — | — | — | — | — | — | 1 |
| 15–19 | 4 | 2 | — | — | — | — | — | 6 |
| 20–24 | 13 | 6 | — | — | 1 | — | — | 20 |
| 25–29 | 34 | 6 | 10 | — | — | — | — | 50 |
| 30–34 | 88 | 27 | 19 | 8 | 1 | 1 | — | 144 |
| 35–39 | 170 | 40 | 29 | 11 | 5 | 4 | 3 | 262 |
| 40–44 | 200 | 37 | 42 | 22 | 5 | 1 | 5 | 312 |
| 45–49 | 188 | 29 | 47 | 19 | 3 | 7 | 2 | 295 |
| 50–54 | 154 | 52 | 39 | 28 | 2 | 5 | 4 | 284 |
| 55–59 | 142 | 30 | 21 | 24 | 2 | 4 | 4 | 227 |
| 60–64 | 98 | 30 | 16 | 32 | 3 | 2 | 4 | 185 |
| 65–69 | 73 | 21 | 17 | 31 | 1 | — | — | 143 |
| 70–74 | 46 | 15 | 9 | 22 | — | 1 | 1 | 94 |
| 75–79 | 20 | 6 | 8 | 13 | 1 | — | — | 48 |
| 80+ | 28 | 5 | 7 | 16 | 2 | — | 1 | 59 |
| Total | 1259 | 306 | 264 | 226 | 26 | 25 | 24 | 2130 |
| Population | 1432456 | 287862 | 156054 | 32588 | 106942 | 92654 | 15341 | 2123897 |
| Crude incidence rate | 9·8 | 11·8 | 18·8 | 77·1 | 2·7 | 3·0 | 17·4 | 11·1 |

TABLE II.—*Average Annual Age-specific and Age-adjusted Incidence Rates among Parsis, Non-Parsis and Total Population, and Mortality Rate in Total Population per* 100,000 *of Female Breast Cancer; Greater Bombay* 1964 *through* 1972

| Age group (years) | Breast cancer (ICD 8th: 174) | | | |
|---|---|---|---|---|
| | Incidence rate/100,000 | | | Mortality in total population rate/100,000 |
| | Parsis | Non-Parsis | Total population | |
| 0–4 | — | — | — | — |
| 5–9 | — | — | — | — |
| 10–14 | — | 0·05 | 0·05 | — |
| 15–19 | — | 0·35 | 0·34 | 0·06 (1)* |
| 20–24 | — | 1·0 | 0·86 | 0·09 (2) |
| 25–29 | — | 2·6 | 2·6 | 0·98 (19) |
| 30–34 | 37·8 | 9·0 | 9·4 | 3·0 (46) |
| 35–39 | 49·5 | 19·9 | 20·5 | 7·1 (91) |
| 40–44 | 90·1 | 32·1 | 33·7 | 11·5 (107) |
| 45–49 | 88·7 | 41·9 | 43·4 | 17·2 (117) |
| 50–54 | 119·0 | 48·5 | 51·5 | 20·9 (115) |
| 55–59 | 138·2 | 61·9 | 65·8 | 29·3 (101) |
| 60–64 | 168·0 | 48·4 | 55·1 | 26·2 (88) |
| 65–69 | 232·4 | 69·7 | 82·2 | 45·4 (79) |
| 70–74 | 171·2 | 58·3 | 68·9 | 35·0 (49) |
| 75–79 | 174·4 | 57·2 | 69·9 | 40·8 (28) |
| 80+ | 215·5 | 102·9 | 119·9 | 103·5 (51) |
| All ages | 77·1 | 10·1 | 11·1 | 4·7 (894) |
| Age-adjusted rate† | 48·5 | 18·2 | 19·9 | 9·2 |

* Figures in parentheses show the number of female breast cancer deaths in 9 years (1964–72).
† Age-adjusted to world population (UICC, 1970).

TABLE III.—*Incidence Rate of Female Breast Cancer by Calendar Year, 1964 through 1972, per 100,000. Average Annual Percentage Change in Incidence, its s.d. and their ratio, t, among Parsis, Non-Parsis and All Communities, Greater Bombay*

| Calendar year | Parsis Age-adjusted rate | Non-Parsis Age-adjusted rate | All communities Crude rate | All communities Age-adjusted rate |
|---|---|---|---|---|
| 1964 | 45·9 | 21·5 | 12·4 | 22·9 |
| 1965 | 49·0 | 15·8 | 9·6 | 17·9 |
| 1966 | 28·6 | 17·8 | 11·0 | 18·3 |
| 1967 | 47·3 | 17·2 | 10·3 | 18·4 |
| 1968 | 66·3 | 18·0 | 11·7 | 20·9 |
| 1969 | 71·2 | 17·1 | 11·0 | 19·5 |
| 1970 | 45·1 | 17·7 | 11·2 | 19·8 |
| 1971 | 43·2 | 16·9 | 10·8 | 18·3 |
| 1972 | 45·1 | 18·8 | 11·6 | 19·7 |
| Average annual % change | 1·46 | 0·58 | 0·31 | 0·50 |
| s.d. | 3·64 | 1·17 | 1·03 | 1·10 |
| t | 0·40* | 0·50* | 0·31* | 0·49* |

* Not significant ($P > 0·05$).

rate of breast cancer in the Parsis is 48·5, while that for women of other religious communities is only 18·2, giving an average of 19·9 per 100,000 for all the religious groups taken together. The average incidence in Parsis is thus 2·7 times higher than in the other Indian communities.

### *Age-incidence time trends*

Table III presents age-adjusted incidence by calendar year. The age-adjusted incidence at this site in the total population has remained steady, between 18 and 23 per 100,000. It appears that the incidence for the year 1964 (the first year of the Registry) was slightly higher, probably because of a spill-over of cases from the preceding years, which were registered for the first time in 1964.

The observed incidence by 10-year age groups (15–24, 25–34, 35–44, 45–54, 55–64 and 65+) is seen to differ between different age groups. At the younger ages, the rates generally do not show any change, whilst in the oldest group they present an irregular trend.

In the Parsis, the observed annual adjusted incidence varies between 28·6 and 71·2, giving an average of 48·5 per 100,000. In women from the other communities the observed incidence rates remain between 15·8 and 21·5, giving an average of 18·2 per 100,000. The average annual changes in incidence rates were not statistically significant, either in the Parsis or in the other communities.

### *Comparison of breast cancer incidence rates in selected countries*

Age-adjusted incidence rates for cancer of the breast at 19·9 per 100,000 for the total population, are thus quite low in Greater Bombay and close to the rates reported (UICC, 1970) from Puerto Rico (20·9) and Yugoslavia–Slovenia (22·8). Populations in South Africa, Bantu (13·6); Japan, Miyagi Prefecture (11·0) and Israel, non-Jews (8·1) present even lower rates than in Greater Bombay. On the other hand, populations in Hawaii, Caucasians (62·9) and U.S.A.–Connecticut (62·3) present 3 times higher rates than Bombay's, except for the Parsi women (48·5) who seem to experience a high incidence of the disease, close to the rate presented by Swedish women (48·6).

### *Mortality*

Registration of deaths in India is generally unsatisfactory, but the position is comparatively better in cities and specifically in the large metropolitan centres of Bombay, Calcutta and Madras, because of reasonably good availability of medical facilities and strict enforcement of rules relating to death registration. In Bombay, in fact, a death registration system has been in operation continuously since 1848. Specifically since 1960, the registration of deaths is quite satisfactory, being complete and reliable. (In Bombay, the doctor/population ratio is approximately 1/750, while for the rest of the country it is 1/5000).

Table II also compares the age-specific morbidity and mortality rates for cancer of the female breast in the total population. To minimize year-to-year variations, these figures have been computed by combining all cases from 1964 to 1972. Both morbidity and mortality data show an increase in incidence rates with age. During the 9-year period, the average annual age-adjusted incidence rate was 19·9, while the mortality remained at 9·2 per 100,000. Thus the case fatality rate from breast cancer is estimated to be 46·2%, which shows that the disease has a high fatality rate in women residents of Greater Bombay.

### COMMENTS

The quality of data in the present publication can be considered satisfactory, as it is derived from sources of known reliability. We do not feel that the results of this investigation could have arisen from any change in the quality of the data or from incomplete registration. In fact, as Greater Bombay is the industrial heart of India, it has reasonably good medical facilities available for the diagnosis and treatment of cancer, which is centralized to a certain extent in the city.

It is therefore unlikely that the observed low incidence, age for age, in the non-Parsis as compared with the Parsi group could have arisen from any bias.

This work was supported in part by Grant NIH-01-006-1 from the National Cancer Institute, Bethesda, Maryland, U.S.A.

### REFERENCES

*Cancer Incidence in Five Continents, Volume I* (1966) Eds. R. Doll, P. Payne and J. Waterhouse. Geneva: International Union Against Cancer.

*Cancer Incidence in Five Continents, Volume II* (1970) Eds. R. Doll, C. Muir and J. Waterhouse. Geneva: International Union Against Cancer.

*Census of India* 1961. *Volume X—Maharashtra* (1964) Part X (1-B) Greater Bombay Census Tables. PRG. 126 (1-B) (Ordinary)/925. Maharashtra, Superintendent of Census Operations.

*Census of India* 1961. *Volume X—Maharashtra* (1971) Part X (1-D) Parsis of Greater Bombay. Bombay: The Maharashtra Census Office.

*Census of India* 1971. *Series* 11, *Part IIA—Maharashtra* (1972) General Population Tables. Maharashtra: Director of Census Operations.

JUSSAWALLA, D. J., HAENSZEL, W., DESHPANDE, V. A. & NATEKAR, M. V. (1968) Cancer Incidence in Greater Bombay: Assessment of the Cancer Risk by Age. *Br. J. Cancer*, **22**, 623.

JUSSAWALLA, D. J. & JAIN, D. K. (1976) *Cancer Incidence in Greater Bombay* 1970-1972*: An Epidemiological Study*. Bombay: Bombay Cancer Registry, Indian Cancer Society.